# Benign and malignant breast lesions in children and adolescents - diagnostic and therapeutic approach

**DOI:** 10.3389/fped.2024.1417050

**Published:** 2024-10-23

**Authors:** Patrycja Sosnowska-Sienkiewicz, Danuta Januszkiewicz-Lewandowska, Przemysław Mańkowski

**Affiliations:** ^1^ Department of Pediatric Surgery, Traumatology and Urology, Poznan University of Medical Sciences, Poznań, Poland; ^2^ Department of Pediatric Oncology, Hematology and Transplantology, Poznan University of Medical Sciences, Poznań, Poland

**Keywords:** biopsy, fibroadenoma, pediatric breast tumor, phyllodes tumor, ultrasound

## Abstract

Benign and malignant breast lesions in children and adolescents are rare compared to adults. Most tumors are benign. Malignant breast lesions are extremely rare. Fibroadenomas are the most common, accounting for 95% of all lesions. Diagnosis is based on history and physical examination of the breast and armpit. Imaging studies include ultrasound, mammography, and magnetic resonance imaging. Ultrasound is the most commonly used imaging test. Other tests are used in cases of diagnostic doubt. Core needle biopsy should be considered for appropriate diagnostic management. Excisional biopsy should be considered for complex clinical conditions and imaging studies. Except in doubtful situations in children and adolescent girls, a conservative approach and observation of the lesions along with periodic ultrasound examination initially every 6–12 months is advisable. Management of malignant breast lesions in children typically involves a multidisciplinary team consisting of pediatric oncologists, surgeons, radiation oncologists, pathologists, and other specialists and depends on the clinical condition of the patient. An important aspect is the experience of the clinician and radiologist in the treatment of breast lesions, as well as increasing patient and family awareness of possible breast lesions and self-examination. This review aims to provide a scoping overview of the available literature on benign and malignant lesions of the breast in pediatric and adolescent populations to assist physicians and surgeons in making decisions regarding the appropriate diagnosis and management of pediatric breast disease.

## Introduction

Breast lesions, especially malignant ones, are rare in children and adolescents compared to adults. The incidence of a breast lesion ranges from 0.1 to 3.25% in the pediatric population, but the literature is limited (1). Benign breast lesions can include conditions such as fibroadenomas, phyllodermal tumors, intraductal papilloma, juvenile papillomatosis, fibrous nodules, tubular adenoma, hamartoma, cysts, fibrocystic lesions or abscess.

Malignant breast lesions can include primary breast neoplasms like breast cancer, malignant phyllodes tumor, metastatic tumors from other primary cancers (rhabdomyosarcoma, neuroblastoma), or infiltration of breast tissue from hematologic malignancies like leukemia or lymphoma (2, 3). Breast cancer in children accounts for less than 1% of all breast cancer cases. The incidence is 0.08 per 100,000 children (4).

Thus, although the diagnosis of a breast lesion may be distressing to parents and the patient personally, the risk in children and adolescents that it is a malignant process is extremely low (3).

This paper summarizes the diagnostic and therapeutic approach for breast lesions in children and adolescents and may be helpful as a regimen for the family physician or pediatrician and pediatric surgeons.

### Diagnostics

Diagnostics for benign and malignant breast lesions in children involve a combination of clinical assessment, imaging studies, and sometimes biopsy for definitive diagnosis. A thorough medical history is obtained, including any symptoms the child may be experiencing, such as the presence of a breast lump, pain, or nipple discharge (1–5).

Physical examination of the breasts is performed to assess for the presence of any palpable masses, nipple abnormalities, skin changes, or lymphadenopathy in the axillary pits. Imaging studies include ultrasonography (USG) and magnetic resonance (MRI). Ultrasonography is often the first-line imaging modality used to evaluate breast lesions in children. It provides detailed images of the breast tissue and can help to differentiate between cystic and solid masses. It is non-invasive making it safe for pediatric patients (6, 7). MRI may be used in certain cases, particularly when additional information is needed to characterize a breast lesion, such as its vascularity and extent (8). Breast Imaging Reporting and Data System lexicon (BI-RADS) is the gold standard for describing and stratifying breast lesions into categories that correlate with the likelihood of malignancy by imaging appearance (3). Most breast lesions in the pediatric population are benign and will be categorized as BI-RADS 2 or 3, emphasizing conservative management. Highly suspicious lesions or lesions in high-risk group children and adolescents require biopsy and are classified in BI-RADS category 4 or higher, but they are extremely rare (3). Table 1 shows BI-RADS assessment categories and risk of cancer.

**Table 1:** BI-RADS assessment categories and risk of cancer. American College of Radiology BI-RADS Atlas 2013.

BI-RADS category	Assessment	Risk of cancer
0	Incomplete – need additional imaging evaluation	Not applicable
1	Negative	Essentially 0%
2	Benign	Essentially 0%
3	Probably benign	>0%, ≤2%
4	Suspicious	>2%, <95%
5	Highly suggestive of malignancy	≥95%
6	Known biopsy-proven malignancy	Not applicable

Fine Needle Aspiration (FNA) biopsy may be performed under ultrasound guidance to obtain a sample from a breast lesion. This procedure is used for cystic lesions to drain fluid and confirm the benign nature of the lesion (3, 8). Core needle biopsy involves the removal of a small tissue sample using a larger needle. It is used to obtain tissue for histological analysis and is performed for solid breast lesions to differentiate between benign and malignant ones (3, 8). In some cases, particularly when biopsy methods are inconclusive or when there is a high suspicion of malignancy, a surgical biopsy may be performed to obtain a larger tissue sample for definitive diagnosis (8). Biopsy should be considered if the mass is larger than 4–5 cm, undergoing rapid enlargement, or based on imaging tests considering the BI-RADS scale to exclude the possibility of a phyllodes tumor (3).

In certain cases, particularly if there is a strong family history of breast cancer or if the child has other risk factors, genetic testing may be recommended (1, 9).

Overall, the diagnostic approach to benign and malignant breast lesions in children involves a multidisciplinary approach, with close collaboration between pediatricians, radiologists, pathologists, genetician and surgeons, to ensure accurate diagnosis and appropriate management (1–6, 8).

### Treatment and prognosis

The treatment of breast lesions in children varies depending on the specific type of lesion, its size, location, characteristics, and whether it is benign or malignant (1). Many benign breast lesions in children, such as fibroadenomas, are observed only in follow-up USG, especially if they are small, asymptomatic, and not growing. Hormonal therapy may be considered to manage certain types of benign breast lesions, such as fibrocystic changes (1–3). Surgical removal may be recommended for benign breast lesions that are causing symptoms (such as pain or discomfort), enlarging, or causing cosmetic concerns. Minimally invasive surgical techniques, such as lumpectomy or excisional biopsy, may be employed to remove the lesion while preserving breast tissue (5).

Therapy for malignant breast lesions differs significantly. Management of malignant breast lesions in children typically involves a multidisciplinary team consisting of pediatric oncologists, surgeons, radiation oncologists, pathologists, and other specialists (4). Surgical resection is often the primary treatment for malignant breast lesions in children. The goal of surgery is to remove the tumor with clear margins while preserving as much healthy breast tissue as possible. Options may include lumpectomy (partial mastectomy) for smaller tumors or mastectomy for larger ones or when preservation of breast tissue is not feasible (3, 4). Chemotherapy may be recommended before or after surgery, depending on the type and stage of the malignant breast lesion (4). Depending on the specific characteristics of the malignant breast lesion, targeted therapy or immunotherapy drugs may be used (4, 10). Regular follow-up care is essential for children treated for malignant breast lesions to monitor for recurrence, manage any treatment-related side effects, and provide ongoing support for the child and their family (1–4).

The prognosis for breast lesions in children varies based on several factors, including the type and characteristics of the lesion, the child's age, overall health, and the effectiveness of treatment. The prognosis for fibroadenomas in children is excellent. Other benign breast lesions, such as cysts and fibrocystic changes, generally have a favorable prognosis (1–3). The prognosis for phyllodes tumor can vary depending on factors such as tumor size, histological subtype, lymph node involvement, and the presence of metastasis. Early detection and prompt treatment are crucial for improving outcomes. With appropriate therapy, including surgery, chemotherapy, and radiation therapy, the prognosis can be favorable. Other types of malignant breast lesions in children, such as metastatic tumors from other primary sites, have varying prognoses depending on factors such as tumor stage, aggressiveness, and response to treatment (11, 12).

Treatment for both benign and malignant breast lesions in children and adolescents may impact breast development, particularly if surgery or radiation therapy is involved. However, efforts are made to minimize cosmetic and functional effects whenever possible (4, 10). Coping with a diagnosis of breast lesions, whether benign or malignant, can have psychosocial implications for children and their families. Supportive care services, including counseling and support groups, may be beneficial to address emotional and social needs (1, 3, 4).

## Detailed characteristics of benign lesions

### Gynecomastia

#### Clinical picture

Gynecomastia typically is localized in the central part of the breast, directly beneath the nipple (areola), as a firm, rubbery, and often disc-shaped mound of tissue. In most cases, gynecomastia is bilateral, but it can be asymmetrical or unilateral in some cases. The enlargement can vary from a small, palpable mass behind the nipple to more prominent breast enlargement (2, 13). Gynecomastia can occur normally during three phases of life. The first occurs shortly after birth in both males and females. This is caused by the high fetal blood levels of mothers estradiol and progesterone and the increased conversion of steroid hormone precursors to sex steroids by luteinizing hormone (LH). Neonatal gynecomastia may persist for several weeks after birth and may be associated with a milky breast discharge. Puberty is the second period when gynecomastia can occur physiologically, and up to 60% of boys have clinically detectable gynecomastia by age 14. Although it is mostly bilateral, it is often asymmetrical and can occur unilaterally. Pubertal gynecomastia usually resolves within 3 years of onset. The mechanism by which pubertal gynecomastia occurs may be due to either decreased production of androgens or increased aromatization of circulating androgens, thus increasing the estrogen to androgen ratio.

Pathologic gynecomastia is due to an increased level of estrogen to androgen in some neoplasms that possess aromatase overactivity (sex-cord tumors, fibrolamellar hepatocellular carcinoma) or because of excess dehydroepiandrosterone (DHEA), DHEA-sulfate (DHEAS), and androstenedione produced by some adrenal tumors. About 20% of gynecomastia is caused by medications or exogenous chemicals and alcohol abuse. Systemic illness, such as hyperthyroidism, liver disease, or renal failure may also be the reason of gynecomastia (2).

#### Diagnostics

The diagnostics of gynecomastia includes medical history, physical examination, laboratory tests, and imaging studies. During the physical examination, palpation of the breast tissue differentiates true glandular enlargement (gynecomastia) from fat deposition (pseudogynecomastia). Pubertal signs such as pubic hair growth, deepening of the voice, or testicular enlargement with gynecomastia are common in early puberty. During the genital examination, the size of the testicles/ovaries and any abnormalities (such as masses or underdeveloped testicles) should be assessed. If gynecomastia is atypical or associated with other symptoms (e.g., rapid onset, pre-pubertal, or associated with other hormonal problems), blood tests may be needed. Hormone levels such as testosterone, estradiol (estrogen), luteinizing hormone (LH), follicle-stimulating hormone (FSH), and prolactin should be measured; thyroid-stimulating hormone (TSH) and free thyroxine (T4) levels assess for hyperthyroidism. Liver and kidney function tests rule out liver or kidney disease that may cause hormonal imbalances. Beta-hCG levels check for testicular tumors, which can produce hormones that cause gynecomastia. In rare cases, a karyotype may be performed if a genetic cause is suspected (e.g., Klinefelter's syndrome) (2). A breast ultrasound can help confirm the diagnosis and differentiate between gynecomastia and other potential causes of breast enlargement, such as tumors or cysts. A testicular/ovarian ultrasound is helpful if there is concern about testicular tumors or abnormalities. In rare and more concerning cases (e.g., unilateral enlargement or suspicious mass), mammography may be used to rule out breast cancer, although this is extremely rare in children (2, 13, 14).

#### Histopathological picture

Gynecomastia is characterized by benign proliferation of ductal and stromal elements in male breast tissue. The histology varies depending on the phase of gynecomastia (early or late). Early (florid) phase reflects active proliferation and is typically seen in recent onset gynecomastia. There is ductal epithelial proliferation with an increase in the number of ducts. The ducts are often elongated and branched. The stroma around the ducts shows edema (swelling) and increased vascularity. Proliferation of fibroblasts (cells that form connective tissue) is present in the periductal stroma. The myoepithelial cell layer underlying the ductal epithelium remains intact, distinguishing gynecomastia from malignant processes. Unlike female breast tissue, lobules are generally absent in gynecomastia, although very rare cases of lobular formation have been described, especially in long-standing gynecomastia.

In long-standing or chronic cases (late, fibrous phase), the tissue shows more fibrotic and less cellular changes. The periductal stroma becomes fibrotic (scar-like tissue), leading to a reduction in the cellularity of the tissue. The number of ducts may be reduced, and the epithelial cells lining the ducts may appear flattened or atrophic. The edema and active fibroblastic proliferation seen in the early phase are no longer present (2, 3, 13).

#### Treatment

A careful breast exam should be done initially every 3–6 months until the gynecomastia regresses or stabilizes, after which a breast exam can be performed yearly (2, 3, 13, 14). If gynecomastia does not resolve on its own after two years, medical treatment is necessary. The options are pharmacotherapy or surgery.

### Fibroadenoma

#### Clinical picture

Fibroadenomas are the most common (95% of all breast lesions) benign breast tumors in adolescents, typically presenting as painless, rubbery lumps found in the breast tissue, often near the surface. It can be in one or both breasts. Fibroadenomas can vary in size, from less than a centimeter to several centimeters in diameter. It tends to move easily under the skin and is not usually fixed to the underlying tissue. Fibroadenomas are typically painless, though occasionally they may cause discomfort or tenderness. In most cases, there are no other accompanying symptoms. However, some individuals may experience breast asymmetry or nipple discharge, though these are less common (1, 3). They are more common in older adolescents but can occur at any age during childhood (2). Fibroadenomas in children are hormonally responsive and may fluctuate in size with hormonal changes during puberty (3, 15).

#### Diagnostics

A thorough physical examination is the first step in diagnosing a fibroadenoma. Ultrasound imaging is commonly used to evaluate breast lumps. If there is any doubt, an MRI scan may be helpful. In some cases, particularly if the diagnosis is unclear based on clinical examination and imaging studies, a fine needle aspiration biopsy may be performed. In situations where a more definitive tissue sample is needed, a core needle biopsy or surgical excisional biopsy may be recommended (1–3, 8, 15).

#### Histopathological picture

They are characterized by a balanced ratio of glandular to stromal proliferation ratio throughout the tumor. The stromal component of the fibroadenoma usually has low cellularity, insignificant nuclear atypia, very low mitotic activity, and no extra growth. The margins of the lesion are usually non-infiltrating. The epithelial component of a fibroadenoma consists of cells derived from the ducts supported by a myoepithelial layer. There are two patterns of epithelial cell growth: intracanalicular and pericanalicular. Both intracanalicular and pericanalicular patterns may coexist in the same lesion. There are several variants of fibroadenoma tumors. These include the usual type/adult, myxoid, complex, cellular, and juvenile fibroadenomas (1).

#### Treatment

##### Observation

In most cases, especially if the fibroadenoma is small, asymptomatic, and confirmed through diagnostic imaging, a wait-and-see approach is recommended. Regular clinical follow-up examinations (every 6–12 months) and imaging studies (USG every 6–12 months, depending on the test result on the BI-RADS scale) may be recommended to monitor any changes in the size or characteristics of the fibroadenoma.

##### Surgical excision

If the fibroadenoma is large, causing discomfort, growing rapidly, or causing significant psychological distress to the child, surgical removal may be recommended. The surgical procedure involves removing the fibroadenoma while preserving as much of the surrounding breast tissue as possible (1–3).

##### Medication

Hormonal therapy, such as oral contraceptives or gonadotropin-releasing hormone agonists, has been used in some cases to reduce the size of fibroadenomas or alleviate symptoms. However, the use of hormonal therapy in children with fibroadenomas is less common and may be reserved for specific situations under the guidance of a pediatric endocrinologist (1–3, 15).

### Phyllodes tumor

#### Clinical picture

Phyllodes tumors are rare lesions, accounting for only 0.3%–0.5% of all breast lesions in adult women, and <10% of these tumors occur in women < 20 years of age (11). Like fibroadenomas, phyllodes tumors typically present as a breast lump. Their cut surface reveals thin, cleft-like spaces that include nodules of stromal growth, and the color of phyllodes tumor gross specimen ranges from tan to yellowish gray (11). Unlike fibroadenomas, phyllodes tumors tend to grow more rapidly and can become quite large. Phyllodes tumors can vary greatly in size, ranging from small to very large masses. Phyllodes tumors may cause pain or discomfort, while other patients may be asymptomatic. Phyllodes tumors have the potential to recur locally after surgical removal, and in rare cases, they can metastasize (spread) to other parts of the body, particularly the lungs (16).

#### Diagnostics

Ultrasound imaging is commonly used to evaluate breast lesions, but MRI scans may be helpful. Histopathologic examination is the only objective means of evaluating suspected phyllodes tumor. FNA biopsy is not a completely reliable method for differentiating between breast fibroepithelial lesions. Surgical excision and histopathologic examination are important to truly differentiate between a fibroadenoma and a phyllodes tumor (1, 3, 17).

#### Histopathological picture

A characteristic leaf-like pattern is formed by intracanalicular proliferation of the stroma and variable lumen sizes of the ducts. Other characteristic features include increased stromal growth and cellularity with increased mitotic activity (18).

The WHO (World Health Organization) has classified phyllodes tumors as benign, borderline, and malignant based on histologic features. Benign tumors are the most common subtype, accounting for 35% to 64% of all phyllodes tumors. Benign tumors have well-demarcated tumor margins, little atypia, low stromal cellularity, <5 mitotic figures per 10 HPF, no stromal overgrowth. Borderline tumors have clear margins or only foci of invasion. Stromal growth is either absent or limited to certain foci. Stromal cellularity is moderate, and moderate nuclear atypia is seen. Malignant lesions account for 25% of all phyllodes tumors and are characterized by markedly increased stromal cellularity and atypia, ill-defined tumor margins, increased stromal growth, and foci of mitotic activity in 10/10 HPFs (1, 3).

#### Treatment

Surgical resection is necessary if a phyllodes tumor is suspected. Depending on the clinical condition, patients diagnosed with malignant phyllodes tumors may even require treatment with mastectomy, chemotherapy, and radiotherapy (16, 17).

### Intraductal papilloma (solitary central papilloma)

#### Clinical picture

Intraductal papillomas are rare benign breast tumors that develop within the milk ducts of the breast. While they are more common in adult women, they can also occur in children, although very rarely. Like in adults, intraductal papillomas in children typically present as a breast lump, small and localized within a milk duct. Intraductal papillomas in children are often unilateral, affecting only one breast. One of the hallmarks of intraductal papillomas is nipple discharge, which may be clear, bloody, or serous. On physical examination, the breast lump associated with an intraductal papilloma may feel small, smooth, and mobile. It is usually located just below the nipple or within the areola. In general, intraductal papillomas are not usually associated with pain. However, if the tumor causes obstruction of a milk duct, it may cause inflammation and discomfort (2, 19).

#### Diagnostics

On ultrasound, a solid intraductal mass is present within a dilated duct filled with anechoic fluid, occasionally with associated vascularity (1–3).

#### Histopathological picture

Histologically, a papilloma is a mass consisting of multiple papillary structures, each defined by a fibrovascular core made up of connective tissue and small blood vessels lined by benign epithelium (2).

#### Treatment

Surgical excision is the treatment of choice because it is necessary to exclude malignancy (3, 19).

### Juvenile papillomatosis (multiple peripheral papillomas)

#### Clinical picture

Juvenile papillomatosis is a rare benign breast condition, which is characterized by the development of multiple papillomas within the breast ducts (in peripheral ducts). This is different from intraductal papilloma, which is a solitary central intraductal papilloma (in a central subareolar duct) (3, 20).

#### Diagnostics

On USG, papillomatosis is seen as ill-defined irregular hypoechoic tissue or masses, occasionally containing cystic spaces. There may be associated clustered microcalcifications. On MRI, papillomatosis presents as lobulated masses with cystic spaces that are well visualized on T2-weighted sequences enhanced with gadolinium (21, 22).

#### Histopathological picture

Histologically, juvenile papillomatosis lacks the fibrovascular core (3).

#### Treatment

Juvenile papillomatosis is a benign condition but is associated with carcinoma in up to 15% of cases. Therefore, surgical resection with margins is indicated to prevent recurrence (3, 20).

### Fibrous nodules

#### Clinical picture

Fibrous nodules usually present as firm palpable masses in premenopausal women but may rarely be seen in the pediatric group. Other names synonymous with fibrous nodule include focal fibrosis, fibrous disease, fibrous mastopathy, fibrosis of the breast, and fibrous tumor (3).

#### Diagnostics

Ultrasound imaging is commonly used, alternatively MRI scans may be helpful (3).

#### Histopathological picture

These lesions show dense fibroconnective tissue like that found in adjacent breast tissue with sparse or absent adipose tissue and the absence of features of other stromal lesions (23).

#### Treatment

A fibrous nodule is an acceptable benign histologic diagnosis for a discrete mass on USG. Periodic imaging surveillance of any mass that meets defined imaging criteria is a reasonable and safe approach. However, if imaging features are suspicious for malignancy, sampling and excision should be considered (23, 24).

### Tubular adenoma

#### Clinical picture

Tubular adenoma of the breast is an epithelial tumor that accounts for 0.13% to 1.7% of all benign breast lesions. It classically occurs in young women and is clinically and imaginarily difficult to distinguish from fibroadenoma. It presents as a painless, well-circumscribed, mobile, slow-growing breast mass without skin or nipple changes (25, 26).

#### Diagnostics

Imaging studies such as ultrasound may be performed to evaluate the breast lump and characterize its features. However, it is difficult to differentiate the lesion from others on imaging studies (25).

#### Histopathological picture

Tubular adenomas are characterized by the presence of well-formed tubules lined by epithelial cells. The lining of the tubules is composed of epithelial cells that may show mild to moderate cytologic atypia. The tubules are surrounded by a fibrous stroma that may contain variable amounts of collagen fibers and myoepithelial cells. The stroma may also show evidence of focal fibrosis or hyalinization. Tubular adenomas typically have a low level of mitotic activity, with few or no mitotic figures seen on histopathologic examination (25).

#### Treatment

Preoperative diagnosis of tubular adenoma is not possible because cytologic examination is not reliable. Only histopathologic examination can provide a definitive diagnosis. For this reason, surgical excision is recommended when there is doubt about possible malignancy or when the size or growth is significant (25, 26).

### Hamartoma

#### Clinical picture

Hamartomas are benign tumors of disorganized mature breast tissue elements. Although this is a relatively common lesion in the adult breast, hamartomas are rare in children and adolescents. Hamartomas can be very large (>10 cm) and can mimic a juvenile giant fibroadenoma. Hamartoma is not associated with any known increased risk of subsequent breast cancer. The clinical presentation is usually a painless mass like fibroadenoma (3).

#### Diagnostics

On ultrasound, hamartomas appear as well-circumscribed oval or round masses that may be hypoechoic, isoechoic, or heterogeneous in echotexture, mimicking the appearance of fibroadenoma. Hamartomas often have a classic “breast within a breast” appearance on mammography due to interspersed areas of radiodense fibroglandular components and radiolucent fatty components within an encapsulated mass (3).

#### Histopathological picture

Histologically, hamartomas present a pseudo-encapsulation and consist of a combination of variable amounts of stromal and epithelial components (3, 27).

#### Treatment

Surgical excision is indicated if the lesion shows rapid progressive growth. Hamartomas may recur if excision is incomplete (3).

### Cysts

#### Clinical picture

Breast cysts in children are rare. The primary clinical feature of breast cysts is the presence of a palpable lump in the breast tissue. The lump may feel round or oval-shaped and may vary in size from small to several centimeters in diameter. Breast cysts usually have a smooth texture and may feel soft or rubbery to the touch. They can move easily under the skin and are usually well-defined. Breast cysts can fluctuate in size during the menstrual cycle, may increase in size and tenderness just before menstruation, and may decrease afterward. In some cases, breast cysts may be associated with nipple discharge. The discharge may be clear, yellowish, or bloody and may occur spontaneously or with pressure on the lump (1, 2).

#### Diagnostics

Ultrasound imaging is used to evaluate breast lesions, but MRI scans may be helpful in the event of difficulty with the differentiation (3).

#### Histopathological picture

A breast cyst is a fluid-filled sac that may be lined with different types of cells depending on its origin and composition. The cyst wall may consist of fibrous tissue, which is typically thin and may or may not show signs of inflammation or scarring. The lining of the cyst wall may be composed of epithelial cells, myoepithelial cells, or both. In cases where the cyst is associated with inflammation or infection, histopathological examination may show signs of acute or chronic inflammation, such as infiltration of inflammatory cells and tissue edema. Chronic or recurrent cysts may show evidence of fibrosis within the cyst wall or surrounding tissue. Depending on the underlying cause of the cyst, histopathological examination may also reveal additional features such as ductal hyperplasia, adenosis, or other proliferative changes in the breast tissue surrounding the cyst (1, 2).

#### Treatment

The treatment of breast cysts in children depends on several factors, including the size of the cyst, the presence of symptoms, and the child's age. Observation is indicated in many cases, especially if the cyst is small, asymptomatic, and confirmed through imaging studies to be benign. This involves regular monitoring of the cyst with clinical examinations and USG. If the cyst is causing discomfort or pain, it may be recommended to alleviate symptoms. Warm compresses applied to the affected breast may also help reduce pain and inflammation. If the cyst is large, causing significant discomfort, or if there is uncertainty about the diagnosis, a procedure called fine-needle aspiration may be performed. It can provide immediate relief of symptoms and may also help confirm the diagnosis by analyzing the fluid for signs of infection or other abnormalities.

In some cases, especially if the cyst recurs frequently or if aspiration alone is not effective, injecting a corticosteroid medication into the cyst may be recommended. Antibiotic therapy may also be helpful. Surgical removal of breast cysts in children is rare and usually reserved for cases where the cyst is large, causing persistent symptoms, or if there is concern about the possibility of malignancy. Surgical excision may involve removing the cyst and some surrounding tissue to ensure complete removal and prevent recurrence (1, 2).

### Fibrocystic changes

#### Clinical picture

Fibrocystic changes in the breasts are known as fibrocystic breast disease or fibrocystic mastopathy. These are common benign conditions characterized by the presence of lumps, cysts, and fibrous tissue in the breast. While fibrocystic changes are more common in adult women, they can also occur in adolescent girls and rarely in prepubertal children. The primary clinical feature of fibrocystic changes is the presence of palpable breast lumps. These lumps may vary in size, consistency, and tenderness. They may be round or oval and may move easily under the skin. Fibrocystic changes may involve the formation of fluid-filled cysts within the breast tissue. The development of fibrous tissue within the breast is also observed and may contribute to the overall lumpiness of the breast. Fibrocystic changes may be associated with breast pain. A nipple discharge is also possible (1, 2).

#### Diagnostics

Ultrasound imaging is commonly used to evaluate breast lesions, but alternatively, MRI scans may be helpful (3).

#### Histopathological picture

Fibrocystic changes are often associated with epithelial hyperplasia, which is an overgrowth of the ductal or lobular epithelial cells. The formation of fluid-filled cysts within the breast tissue is observed. These cysts are typically lined with epithelial cells and filled with proteinaceous fluid. Fibrosis may be seen in the stroma surrounding the ducts and lobules. Adenosis may be associated with epithelial hyperplasia (1–3).

#### Treatment

In most cases, fibrocystic changes in the breasts in children do not require invasive treatments or interventions. Regular follow-up appointments with USG examinations are recommended. If symptoms are severe or significantly affect quality of life, hormonal management strategies such as oral contraceptives or hormone therapy may be considered (1–3).

### Abscess

#### Clinical picture

Mastitis and abscesses can occur in childhood. Mastitis occurs in infants and in later childhood (8–17 years) and is thought to be related to skin infection and/or ductal obstruction. Typically, purulent mastitis presents with edema and erythema, occasionally with fever and leukocytosis. Prolonged mastitis may lead to the development of phlegmon and abscess. The most common pathogen is Staphylococcus aureus (3).

#### Diagnostics

Ultrasound should be performed in patients with clinical symptoms of infection to rule out the presence of a fluid collection that can be drained (3).

#### Histopathological picture

Histopathologic examination reveals purulent material within a cavity or space in the breast tissue.

#### Treatment

Treatment includes antibiotic therapy and prompt ultrasound-guided drainage, which facilitates healing and provides culture and susceptibility material for further management (3).

## Detailed characteristics of malignant lesions

### Malignant phyllodes tumor

The characteristics of the malignant form of phyllodes tumor is presented and differentiated above along with those of the benign form.

### Metastatic tumors from other primary sites

#### Clinical picture

Compared to primary breast neoplasms, metastatic disease is more common. Primary tumors that are known to metastasize to the breast in children include renal cell carcinoma, rhabdomyosarcoma, neuroblastoma, Burkitt lymphoma, leukemia, Ewing sarcoma, and melanoma. Metastatic disease in the breast can be single or multiple lesions involving one or both breasts (3, 28).

#### Diagnostics

An enlarging breast mass in a child with a known history of primary malignant neoplasm warrants sampling, even if it looks benign on imaging. Ultrasound may show a circumscribed or irregularly marginated mass with heterogeneous or hypoechoic echo texture. When in doubt, MRI or Positron Emission Tomography- Computed Tomography (PET-CT) may be helpful (3, 28).

#### Histopathological picture

The primary neoplasm determines the histopathological picture (3).

#### Treatment

The primary neoplasm determines the treatment (3, 28).

### Invasive ductal carcinoma

#### Clinical picture

Primary breast cancer incidence is noted to be approximately 0.1 case per million in females younger than 20 years of age (29). The most common subtype of invasive breast cancer in children is invasive secretory carcinoma (ISC), which has a more favorable prognosis compared to the less common subtypes of breast cancer in children (invasive ductal, invasive lobular, medullary, inflammatory, and anaplastic carcinomas). While breast cancer in children is exceptionally rare, ISC accounts for a very small proportion of pediatric breast cancers. ISC in children may present similarly to ISC in adults, with the presence of a breast lump, typically small circumscribed painless palpable masses (usually <3 cm), and nipple discharge (29, 30).

#### Diagnostics

On ultrasound, they typically appear as a circumscribed or partially microlobulated hypoechoic mass with a heterogeneous internal echo texture. A more accurate diagnosis is MRI. After an ultrasound/MRI examination, a patient may be qualified for a breast biopsy.

#### Histopathological picture

The histopathological picture of breast cancers in children does not differ from that in adults.

#### Treatment

Treatment consists of surgical excision, sentinel node biopsy, and possible systemic adjuvant chemotherapy, depending on the extent of the disease. Radiation therapy is possible as a form of therapy but is not recommended in the pediatric group (29).

### Other

The increased risk of breast cancer in patients who have undergone radiation therapy due to primary malignancy and hereditary forms of breast cancer should also be considered (3).

**Figure 1:**
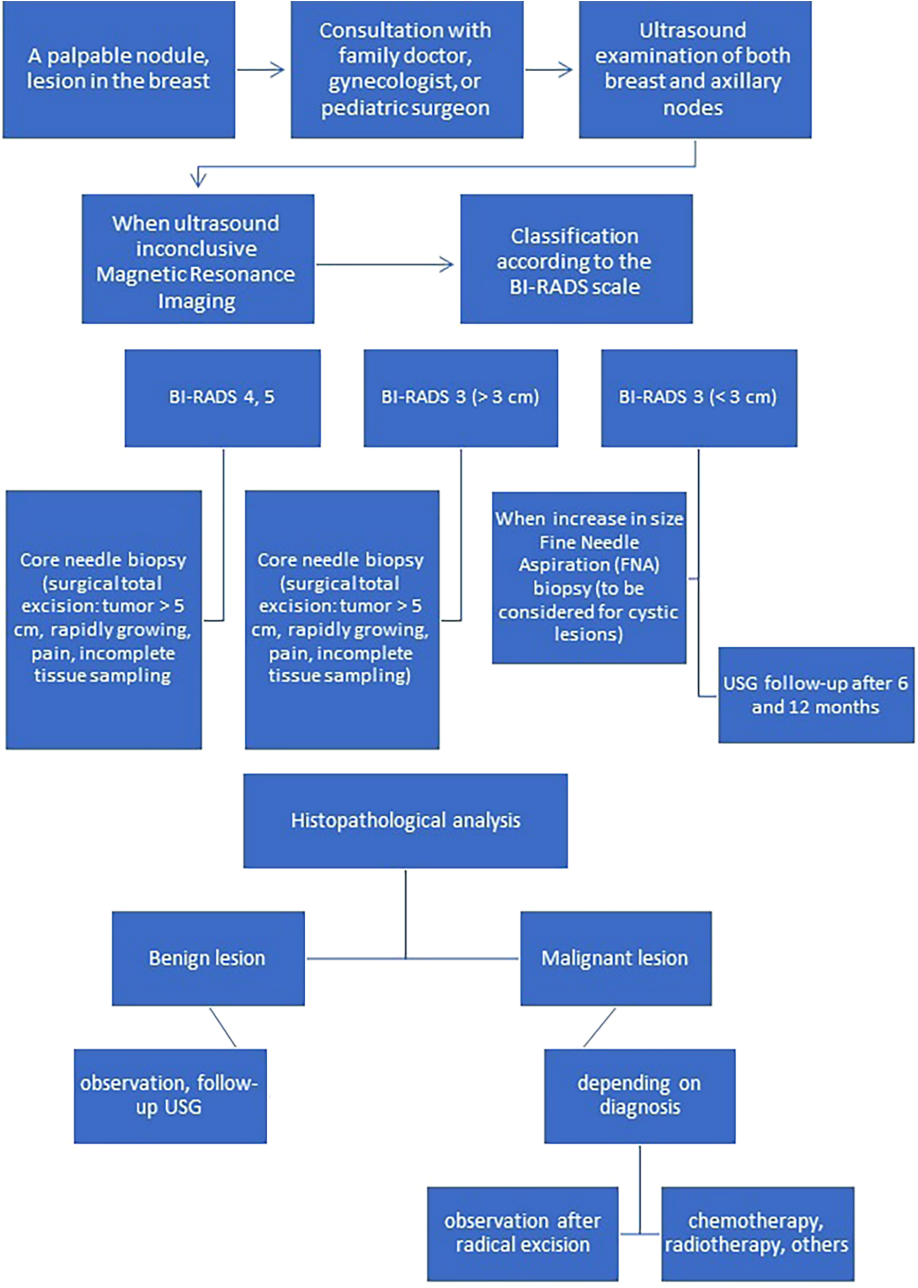
Algorithm for appropriate diagnosis and management of breast lesions in children and adolescents.

A summary of the clinical picture, diagnostic and therapeutic management of benign and malignant tumor breast lesions in the pediatric population is presented in Table 2 and on Figure 1 (2, 3).

**Table 2:** Clinical picture, diagnostic and therapeutic management of benign and malignant tumor breast lesions in the pediatric population.

Diagnosis	Clinical picture	Diagnostics	Treatment
Benign lesions			
Gynecomastia	Firm, rubbery, and often disc-shaped mound of tissue; uni- or bilateral.	USG, blood examination.	Observation/Pharmacotherapy/Surgical excision.
Fibroadenoma	Painless, rubbery lumps found in the breast tissue, often near the surface; uni- or bilateral.	USG MRI and core needle biopsy in case of doubt	Observation with USG follow-up every 6–12 months Surgical total excision if: - tumor >5 cm, - rapidly growing, - causing pain
Phyllodes tumors (benign)	Lump; tendency to grow rapidly and be large.	USG MRI may be helpful FNA (may be unreliable)	Surgical excision with USG follow-up every 6–12 months.
Intraductal papilloma	Small, smooth, and mobile lump; usually located just below the nipple or within the areola; often unilateral; symptoms of inflammation possible.	USG MRI may be helpful	Surgical excision with USG follow-up every 6–12 months.
Juvenile papillomatosis	Solitary, well-defined, mobile breast mass; usually unilateral.	USG MRI may be helpful	Surgical excision with USG follow-up every 6–12 months.
Fibrous nodules	Firm palpable masses.	USG MRI may be helpful	Observation with USG every 6–12 months. If any suspicious for malignancy, sampling and excision should be considered.
Tubular adenoma	Painless, well-circumscribed, mobile, slow-growing breast mass without skin or nipple changes.	USG MRI may be helpful	FNA biopsy (may be unreliable). Surgical excision is recommended.
Hamartoma	Painless mass; large diameter is possible.	USG MRI may be helpful	Observation with USG every 6–12 months. Surgical excision if the lesion showing rapid progressive growth.
Cysts	Palpable lump, round or oval-shaped; usually with a smooth texture and soft or rubbery to the touch.	USG	Observation with USG every 6–12 months. FNA biopsy and fluid aspiration with antibiotic therapy. Surgical excision- rare.
Fibrocystic changes	Palpable breast lumps; vary in size, consistency, and tenderness.	USG	Observation with USG every 6–12 months. Severe symptoms- oral contraceptives or hormone therapy.
Abscess	Painful mass with edema, erythema, occasionally with fever.	USG, blood examination.	Antibiotic therapy and/or ultrasound-guided drainage.
Malignant lesions			
Phyllodes tumor (malignant)	Lump; tendency to grow rapidly and be large.	USG/MRI Core needle biopsy FNA biopsy (may be unreliable).	Wide local excision with 1 cm margins. For recurrent tumor mastectomy, radiotherapy.
Metastatic tumors from other primary sites	Single or multiple lumps; uni- or bilateral.	USG/MRI/PET-CT Core needle biopsy, surgical biopsy or surgical excisional biopsy.	Treatment acc. to protocol for primary tumor.
Invasive ductal carcinoma	Lump, typically small circumscribed painless palpable masses (usually <3 cm), with nipple discharge.	USG/MRI FNA biopsy Core needle biopsy.	Surgical excision, mastectomy, sentinel node biopsy; adjuvant chemotherapy and radiotherapy.

## Discussion

The incidence of benign and malignant breast lesions in children and adolescents is notably rarer than in adults. Although the number of publications on breast changes in the pediatric age group has increased in recent years, the knowledge is still limited (31). It should be stressed that the knowledge of the normal USG/MRI imaging features during child development by radiologists is a very important point for the diagnosis of breast lesions in children and young adults. Radiologists should recognize the differences between children and adults. In children and adolescents, almost all breast tumors are benign (1–3, 9). The most common histopathologic diagnoses are fibroadenoma and benign phyllodes tumor (9, 32). FNA biopsy is not a completely reliable method for differentiating between breast fibroepithelial lesions (9). McLaughlin et al. reported on 196 patients under the age of 18 who underwent excision of a breast mass at their institution between 2008 and 2016. After surgical resection, 3% of benign phyllodes tumors were identified on histopathologic examination. Prior to resection, all patients underwent FNA biopsy, which revealed a phyllodes tumor in only 1 patient, which was later reclassified as a fibroadenoma (32). Trucut biopsy may be necessary to determine strategy prior to surgery because, although extremely rare, primary malignant lesions of the breast are possible (9). Mubarak et al. suggest that in cases where breast lesions cannot be differentiated as fibroadenoma or phyllodes tumor, the next step in evaluation is core needle biopsy (1). Englert et al. concluded that pediatric patients should not be subjected to unnecessary needle biopsies based on a remote family history of breast cancer. They reported that breast masses in the pediatric and adolescent population can be safely managed with regular sonographic evaluation and are concerned about iatrogenic damage to the developing breast (33). Gao et al. emphasize a different approach. They suggest that definitive diagnosis of phyllodes tumors may be difficult with core biopsy, often necessitating surgical excision for complete pathologic evaluation (3). Since most of the lesions are benign, a cautious and expectant approach is recommended in children and adolescents. Surgical excision remains indicated for rapidly growing tumors, symptomatic breast mass, tumor >5 cm regardless of benign ultrasound features, or initially benign pathology on biopsy, because phyllodes tumor cannot be excluded (1, 3).

Considering that lesions in the pediatric group are predominantly benign, the prognosis for such lesions is good. Patients who have mutations in genes including TP53, BRCA1, BRCA2, CDH1, PALB2, ATM, or have an inherited syndrome such as Peutz-Jeghers syndrome, PTEN, Hamartoma tumor syndromes, Neurofibromatosis type 1 are at a higher risk of developing malignancy, so this cohort of patients may need an earlier qualification for biopsy or removal of the lesion (34). Human race, or ethnicity, can influence the incidence of breast tumors through a complex interplay of genetic, environmental, and socioeconomic factors (35). Certain ethnic groups carry genetic mutations that predispose individuals to a higher risk of breast cancer. For example, Ashkenazi Jewish women have a higher prevalence of BRCA1 and BRCA2 mutations, which significantly increase their risk of developing breast cancer (35). Differences in hormonal and reproductive factors across ethnic groups may also affect breast cancer risk. For example, early onset of menstruation and late menopause are associated with increased breast cancer risk (35). Socioeconomic status can affect access to health care, screening programs, and lifestyle factors such as diet and physical activity, which in turn affect breast cancer risk. Environmental exposures, such as pollution, diet, and lifestyle choices, can vary by ethnic group and contribute to differences in breast cancer incidence (35). Due to the small number of cases of breast cancer in children and adolescents, knowledge of factors that specifically affect the occurrence of lesions in children and adolescents is sparse (9, 36).

In conclusion, there are insufficient clinical data regarding the management of breast lesions in children and adolescents. It is prudent to treat breast lesions as benign tumors and adopt a watchful waiting approach. However, in the presence of worrisome features, excisional biopsy of the lesion should be considered for reliable histopathologic evaluation. When evaluating breast lesions in children and adolescents, the experience of the clinician and radiologist is extremely important, and it is necessary to increase patient and family awareness of physiologic breast development and breast self-examination (9).

## Conclusion

Breast lesions in children and adolescents are rare, especially malignant ones. Despite the benign nature of most lesions, the correct diagnostic and therapeutic process must be carried out with due caution, considering the physiological development of the mammary gland on the one hand and the risk of malignant lesions on the other. A special group of patients are those with a positive family history of breast cancer and patients with a previous history of cancer. In children and adolescents, breast lesions most often require a conservative approach and periodic ultrasound follow-up. If there is any doubt about the nature of the lesion, it may be necessary to expand the diagnosis, including magnetic resonance imaging, needle biopsy, or excisional biopsy. An important aspect is the experience of the clinician and radiologist in the treatment of breast lesions, as well as increasing patient and family awareness of possible breast lesions and self-examination.
